# Association of increased oncostatin M with adverse left ventricular remodeling in patients with myocardial infarction

**DOI:** 10.5937/jomb0-37150

**Published:** 2022-10-15

**Authors:** Anna M. Gusakova, Tatiana E. Suslova, Maria A. Kercheva, Irina V. Kologrivova, Tamara R. Ryabova, Vyacheslav V. Ryabov

**Affiliations:** 1 Russian Academy of Science, Tomsk National Research Medical Centre, Cardiology Research Institute, Tomsk, Russian Federation

**Keywords:** pro-inflammatory cytokines, oncostatin M, myocardial infarction, adverse left ventricular remodeling, proinflamatorni citokini, onkostatin M, infarkt miokarda, neželjeno remodeliranje leve komore

## Abstract

**Background:**

The study of laboratory biomarkers that reflect the development of adverse cardiovascular events in the postinfarction period is of current relevance. The aim of the present study was evaluation of oncostatin M (OSM) concentration changes in the early and late stages of myocardial infarction and evaluation of the possibility of its use in prediction of adverse left ventricular (LV) remodeling in patients with myocardial infarction with ST-elevated segment (STEMI).

**Methods:**

The study involved 31 patients with STEMI admitted in the first 24 hours after the onset of MI and 30 patients with chronic coronary artery disease as a control group. Echocardiographic study was performed on day 3 and in 6 months after STEMI. The serum levels of biomarkers were evaluated on the day of hospital admission and 6 months after MI using multiplex immunoassay.

**Results:**

OSM level increased during the first 24 h after the onset of the disease, with the following decrease in 6 months. OSM concentration at admission had correlated with echocardiography parameters and Nt-proBNP, troponin I, CK-MB levels. Our study has demonstrated association of the increased levels of OSM at the early stages of STEMI with development of the adverse LV remodeling in 6 months after the event.

**Conclusions:**

Elevation of OSM levels in the first 24 h after STEMI is associated with the development of the adverse LV remodeling in the long-term post-infarction period.

## Abstract (sr)

**Uvod:**

Proučavanje laboratorijskih biomarkera koji odražavaju razvoj neželjenih kardiovaskularnih događaja u periodu nakon infarkta je trenutno relevantno. Cilj ove studije bio je procena promene koncentracije onkostatina M (OSM) u ranim i kasnim stadijumima infarkta miokarda i procena mogućnosti njegove upotrebe u predviđanju neželjenog remodeliranja leve komore (LV) kod pacijenata sa infarktom miokarda sa ST-povišenim segmentom (STEMI).

**Metode:**

U istraživanju je učestvovao 31 pacijent sa STEMI primljenim u prva 24 sata od pojave IM i 30 pacijenata sa hroničnom koronarnom bolešću kao kontrolna grupa. Ehokardiografska studija je urađena 3. dana i 6 meseci nakon STEMI. Nivoi biomarkera u serumu su procenjeni na dan prijema u bolnicu i 6 meseci nakon IM primenom multipleksnog imunoeseja.

**Rezultati:**

Nivo OSM je porastao tokom prva 24 h od pojave bolesti, sa sledećim smanjenjem za 6 meseci. Koncentracija OSM pri prijemu je bila u korelaciji sa parametrima ehokardiografije i nivoima Nt-proBNP, troponina I, CK-MB. Naša studija je pokazala povezanost povećanih nivoa OSM u ranim fazama STEMI sa razvojem neželjenog remodeliranja LV u 6 meseci nakon događaja.

**Zaključak:**

Povišenje nivoa OSM u prva 24 h nakon STEMI je povezano sa razvojem neželjenog remodeliranja LV u dugotrajnom postinfarktnom periodu.

## Introduction

Myocardial infarction (MI) remains the leading cause of mortality and morbidity all over the world. Patients who suffered MI with ST-elevated segment (STEMI) have an increased risk of sudden death and recurrent events in the future, mainly due to the subsequently developing heart failure (HF) both with the preserved and reduced ejection fraction [1] [2].

Even though modern therapeutic approaches allowed to significantly improve management of STEMI patients, the post-infarction HF still represents a challenge in the modern medicine [3]. Decompensation of the congestive HF represents one of the major causes of mortality in STEMI patients [4] and leads to the re-admission to the hospital in 20% of cases [5] [6]. The prior myocardial infarction is known to initiate the processes of structural rearrangement of myocardium, leading to the development of postinfarct-remodeling. The underlying pathophysiological basis for adverse myocardial remodeling is infarct expansion. The degree of infarct expansion depends upon the size of infarction, wall stress in the left ventricle and improper myocardial healing [7]. Myocardial healing is the least manageable process in this triad, known to be dependent upon regulation of inflammation and its resolution.

Neutrophils and monocytes/macrophages are recruited to the injured myocardium, performing phagocytosis of the necrotic tissue meanwhile releasing mediators of inflammation, such as leukotrienes, thromboxane, prostacyclin, which may increase tissue damage even further [8]. Myocardial necrosis leads to activation of the compliment system, production of the reactive oxygen species and initiates cytokine cascade and acute phase proteins' production by endothelial cells, monocytes and macrophages. The size of injury determines the power of inflammatory response, while impairments in the synthesis of inflammatory mediators may affect the success of the myocardial healing and patients' prognosis. Cytokines synthesized in the focus of inflammation, influence all the cells, responsible for the proper myocardial remodeling, including cells of the innate and adaptive immunity, fibroblasts and endotheliocytes [9]. With all the variety of the known cytokines involved in postmyocardial inflammation and healing, biomarkers, allowing to predict whether the process of resolution of inflammation and myocardial remodeling in a definite patient will be successful, are absent.

Cytokines of IL-6 family are known to play an important role in initiation, sustainment and resolution of the local and systemic inflammation [10] [11]. Pleiotropic cytokine oncostatin M (OSM) is secreted by T cells, monocytes/macrophages, dendritic cells and neutrophils and is known to be tightly involved in the pathogenesis of cardiovascular disorders [12] [13] [14] [15] [16] [17]. Among the biological functions of OSM are regulation of proliferation, inflammation, cellular differentiation, apoptosis and regeneration of various tissues [12] [13] [14] [15] [16] [17] [18] [19] [20] [21]. OSM was elevated in patients with significant and more severe coronary stenosis [22]. On the other hand, OSM appeared to favor post-infarct myocardial healing, possibly through the potentiation of cardiomyocytes dedifferentiation and autophagy [15] [17]. The impact on OSM on heart remodeling appeared to be even more complex when it became clear that it may be either healing or detrimental depending on the nature of myocardial injury: chronic or acute [19]. It is known that OSM is elevated in patients with heart failure with reduced left ventricular ejection fraction [23], however there are no data how the level of OSM at the early phase of STEMI may influence this process.

In summary, currently there are no consistent results indicating the role and prognostic value of OSM in left ventricular post-infarct remodeling and its relationships to the widely used biomarkers of heart injury and stress. And the existing data were mainly acquired in animals and have not yet been properly translated into clinics.

Thus, the aim of the present study was evaluation of OSM concentration changes in the early and late stages of myocardial infarction and evaluation of the possibility of its use in prediction of adverse left ventricular remodeling in the late post-infarct stage in patients with acute anterior STEMI.

## Materials and Methods

### Study patients

In total 31 patients with primary anterior STEMI (58.5±8.5 y.o.), admitted to the intensive care unit during the first 24 hours after the onset of myocardial infarction, have been recruited to the study. Patients with poor visualization of the heart, acute LV dysfunction (Killip class III-IV), sinus bradycardia, permanent atrial fibrillation, valvular heart disease, HF decompensation (NYHA class III-IV), severe concomitant pathology, refusal to participate and patients older than 75 y.o. were excluded from the study. The control group is recruited from patients with chronic coronary artery disease (CAD) (n=30). The protocol of the study was approved by the local ethic committee (protocol #116 from 30.01.2014), and it was developed in compliance with the Medical Association Declaration of Helsinki »Ethical principles for medical research involving human subjects«. All patients signed an informed consent forms prior to participation in the study.

### Echocardiography

Echocardiography was performed in all patients on day 3 and in 6 months after STEMI. Quantification was performed according to the recommendations of the American and European Associations of Echocardiography. End diastolic and systolic volumes (ESV), and left ventricular ejection fraction (LV EF) were determined using Simpson's method. To assess post infarction myocardial remodeling, the dynamics of ESV was evaluated on day 3 of hospital admission and in 6 months after MI. The widely excepted approach to acknowledge the presence of the adverse left ventricular remodeling is an observed increase of left ventricular end-diastolic (and/or end-systolic) ventricular volume (LVEDV and/or LVESV) by at least 20% since the first post-myocardial measurement [24]. Hence the patients were divided into groups based on the results of echocardiography: group 1 – patients with no LV remodeling (∆ESV <20%); group 2 – patients with adverse LV remodeling (∆ESV 20%).

### Serum biomarkers

Venous blood was drawn into sterile vacutainers at admission to the clinics and in 6 months after STEMI. Obtained blood samples have been stored at the room temperature for 30 min and were centrifuged at 3000 rpm for 15 min. The serum samples have been collected and stored at –40 °C until the measurements were performed.

The levels of OSM, troponin I, N-terminal fragment of brain natriuretic peptide (Nt-proBNP) and creatine phosphokinase cardiac specific isoenzyme MB (CK-MB) were evaluated in serum using multiplex immunoassay with the FLEXMAP 3D System (Luminex Corporation, USA) and the Human Cardiovascular Disease Panel 1 (Merck KGaA, Darmstadt, Germany). The study was conducted using the Core Facility »Medical genomics«, Tomsk NRMC.

### Statistical analysis

The analysis of the obtained data was performed using the STATISTICA 10.0 and SPSS 10.0 software. The type of the distribution of the data was evaluated by Kolmogorov-Smirnov test. Results were presented as mean ± standard deviation (x̅ ± SD) in the case of normal distribution and as the median value and the interquartile range (Me (Q1; Q3)) in the case of non-normal distribution. Wilcoxon test was used to estimate the significance of differences in the values of the dependent parameters. The Mann-Whitney U-test was used to estimate the significance of differences between independent groups. The Spearman's rank correlation coefficient was calculated to assess the relationship between the parameters. A value of p<0.05 was considered statistically significant in all statistical evaluations. Linear regression model was created to evaluate input of OSM in the development of the left ventricle adverse remodeling. The prognostic value was determined using the ROC analysis. The area below the ROC curve was calculated, and the sensitivity and specificity were calculated to evaluate the characteristic curves.

## Results

Clinical characteristics of STEMI and chronic CAD patients were comparable (Table 1).

**Table 1:** Clinical and anamnestic characteristics of patients with acute STEMI and chronic CAD Abbreviations: STEMI, myocardial infarction with ST-elevated segment; CAD, coronary artery disease; p, the level of statistical significance of differences between the STEMI patients and CAD patients

Parameter	STEMI patients<br> (n = 31)	Chronic CAD patients<br> (n = 30)	p
Age, years	59.3 ± 8.4	60.4 ± 7.1	0.606
Male, gender, n (%)	23 (74)	19 (63)	0.289
Obesity, n (%)	21 (68)	25 (83)	0.359
Hypertension, n (%)	24 (77)	27 (90)	0.748
Diabetes mellitus, n (%)	8 (26)	9 (30)	0.531
Dyslipidemia, n (%)	22 (71)	18 (60)	0.812

The serum levels of all studied biomarkers in STEMI patients at admission were increased compared to patients with chronic CAD (Table 2).

**Table 2:** Serum concentration of biomarkers in patients with STEMI and chronic CAD Abbreviations: pT1-T2 – the level of statistical significance of differences between STEMI patients at admission and in 6 months; p*– the level of statistical significance of differences between the STEMI patients at admission and CAD patients; p**– the level of statistical significance of differences between the patients in 6 months after STEMI and CAD patients

Biomarker	Patients with STEMI (n = 31)		Patients with chronic CAD<br>(n = 30)	pT1-T2	p*	p**
	Hospital admission<br> (T1)	6 month after<br>STEMI (T2)				
OSM, ng/L	46.90<br>[18.91; 75.73]	13.12<br>[6.88; 19.58]	4.35<br>[1.48; 12.63]	< 0.001	< 0.001	0.006
Troponin, μ g/L	147.48<br>[18.65; 431.18]	0.06<br>[0.03; 0.21]	0.19<br>[0.07; 0.33]	< 0.001	< 0.001	0.137
Nt-proBNP, ng/L	1127<br>[467.02; 1611]	325.64<br>[235.65;554.53]	381.19<br>[220.91; 536.12]	0.022	0.002	0.583
CK-MB, μ g/L	213.63<br>[125.16; 239.69]	2.85<br>[1.88; 3.13]	3.39<br>[2.07; 5.22]	< 0.001	< 0.001	0.101

Only OSM remained elevated in STEMI patients in 6 months of observation compared to chronic CAD patients (Table 2).

We have revealed the significant decrease of the OSM concentration in the long-term post-infarct period (6 months of observation) compared to the elevated values at admission (Table 2). Other biomarkers (troponin I, Nt-proBNP, CK-MB) were also considerably elevated at admission and significantly decreased in 6 months (Table 2).

**Figure 1:**
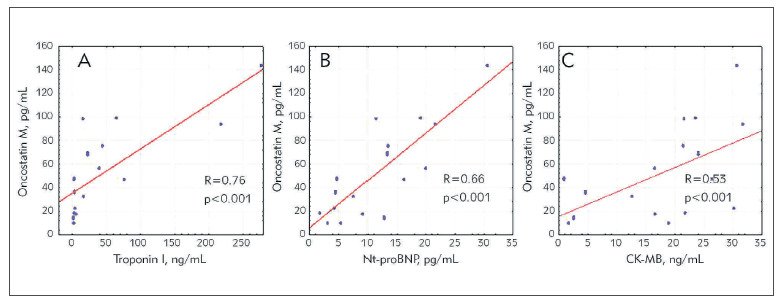
Correlations between oncostatin M and markers of myocardial injury, myocardial stress and necrosis in patients with acute primary STEMI at the first 24 hours from the onset of the disease A – Troponin I, B – N-terminal fragment of brain natriuretic peptide Nt-proBNP, C- creatine phosphokinase cardiac specific isoenzyme MB, CK-MB

The results of correlation analysis demonstrated strong direct relations between OSM and widely acknowledged biomarkers of myocardial injury, stress and necrosis: troponin I, Nt-proBNP and CK-MB (Figure 1). We have also observed correlations between OSM and troponin I in 6 months of follow-up (R = 0.751, p = 0.0005).

**Figure 2:**
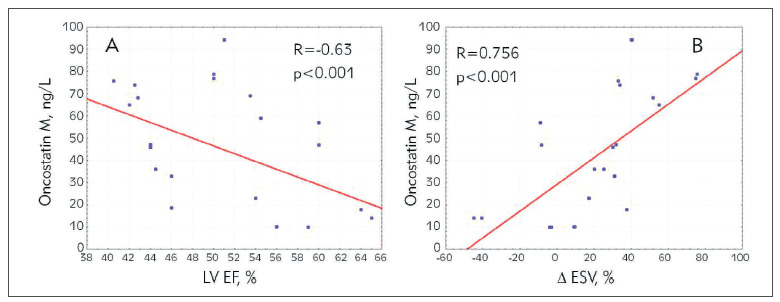
Correlations between oncostatin M at the first 24 hours from the onset of the disease and echocardiography parameters in STEMI patients A – left ventricular ejection fraction (LV EF) value 6 months after MI, B – the increased end systolic volumes (ΔESV)

During analysis of interactions between concentrations of biomarkers and long-term echocardiographic data we revealed negative correlations between OSM concentration at admission and EF LV in 6 months of observation after STEMI (Figure 2A). Concentration of OSM at admission was also negatively related to the values of increase of the LV endsystolic volume (∆ESV) (Figure 2B).

The total group of patients was divided into 2 groups: group 1 – patients without LV remodeling (∆ESV<20%); group 2 – patients with adverse LV remodeling (∆ESV 20%). Medical history and clinical characteristics of patients depending on the presence of the adverse LV remodeling are represented in (Table 3).

**Table 3:** Medical history and clinical characteristics of patients with acute STEMI depending to the development of left ventricular adverse remodeling Abbreviations: EDV, end diastolic volumes; ESV, end systolic volumes; LVEF, left ventricular ejection fraction; HF, heart failure; MI, myocardial infarction

Parameter	Group 1 (n = 17)	Group 2 (n = 14)	p
Killip class at the admission, 1 / 2 / 3 / 4, n	12 / 3 / 1/ 1	10 / 3 / 1 / -	0.810
Preinfarction angina, n	8	8	0.164
Reperfusion time (hours)	5.2 ± 2.2	4.8 ± 2.8	0.236
Complete revascularization, n (%)	11 (64)	8 (57)	0.241
1/2/3 vascular lesion coronary arteries, n	15 / 0 / 1	9 / 5 / 0	0.134
Thrombolysis + percutaneous coronary intervention, n (%)	10 (59)	7 (50)	0.257
Time from pain onset-PCI center (hours)	4.2 ± 2.5	4.1 ± 3.2	0.217
LV EDV (3d day), mL	115.0 ± 19.7	89.6 ± 21.2	0.151
EDV (day 3, 6 month, %)	-2.5 [-14.3; 9.8]	-21.3 [-34.5; -5.7]	0.000
LV ESV (3d day), mL	53.6 ± 12.5	37.7 ± 10.4	0.016
ESV (day 3, 6 month, %)	-3.1 [-17.3; 8.3]	-33.9 [-51.9; -25.4]	0.000
LV EF (3d day), %	53.0 ± 8.7	57.1 ± 10.4	0.104
LV EF (6 month), %	55.1 ± 8.8	51.8 ± 8.3	0.012
HF NYHA >1 at the discharge, n	0	1	0.873
Angina pectoris FC III, n	1	2	0.351
Recurrent MI, n	0	1	0.599
Dead, n	0	0	-

The comparative analysis demonstrated that patients in group 2 had significantly elevated values of ∆EDV and ∆ESV in 6 months, ESV LF at day 3 and LV EF in 6 months compared to patients in group 1 (Table 3).

**Figure 3:**
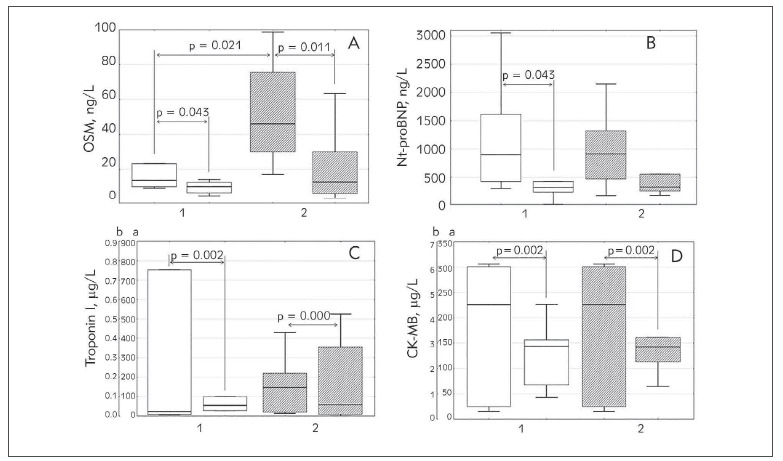
The biomarkers dynamics in patients with STEMI A – oncostatin M, B – Nt-proBNP, C – troponin I, D – CK–MB; Comments: 1, group 1 (ΔESV less than 20%); 2, group 2 (ΔESV more than 20%); scalea, for biomarkers values obtained in 1st day, scale b, for biomarkers values obtained in 6 month after MI

We have also revealed significant differences in the level of OSM at admission in groups 1 and 2 (Figure 3). Concentration of OSM decreased in both groups in 6 months, being comparable in groups 1 and 2 (Figure 3).

Even though Troponin I and CK-MB have been shown to reflect the infarct size and hence predict the development of adverse remodeling [24], we did not detect the statistically significant difference of these biomarkers concentrations in two groups. The level of NT-proBNP was also comparable in groups 1 and 2 (Figure 3), probably due to the wide spread of values.

We have created the model of the paired logistic regression with binary dependent variable to evaluate input of OSM in the development of the adverseLV remodeling. The dependent variable had taken the value «1» if a patient should have been assigned to the group 1, and value »2« if a patient should have been assigned to the group 2. The model employed OSM as a predictor. Logistic regression demonstrated that elevated concentration of OSM at admission was associated with adverse LV remodeling in 6 months (95% CI, 0.57–0.87; p< 0.05). The logistic regression model had the level of the statistical significance p=0.002, the Chi-square = 13.965. The parameters of the logistic regression are represented in Table 4.

**Table 4:** Parameters of the logistic regression model Abbreviations: OSM, oncostatin M; CI, confidence interval

Parameters	Constant B	OSM
Estimate	-1.87	0.077
Standard Error	0.954	0.031
Wald’s Chi-square	3.832	6,185
-value	0.051	0.013
Odds ratio	0.155	2.079
-95% CI	0.022	2.014
+95% CI	1.084	2.149

The ROC analysis showed that the OSM measured at admission to the hospital is a promising informative indicator for predicting an increase in LVESV in 6 months after MI.

**Figure 4:**
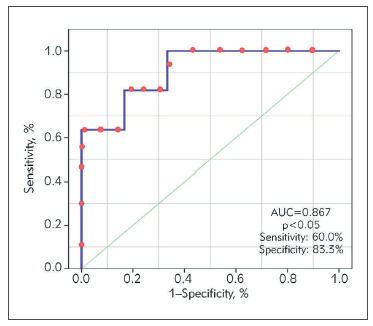
ROC-curve for assessing the adverse remodeling in patients with STEMI Abbreviations: AUC, area ROC-curve

The area below the ROC curve was 0.867 (p<0.05), which made it possible to classify it as »of high quality« (Figure 4). The optimal sensitivity/specificity ratio was 83.3 and 60%, respectively. The OSM value exceeding 18.40 ng/L was associated with an increase in ESV in the long-term post infarction period by more than 20%.

## Discussion

Our study has demonstrated association of the increased levels of OSM at the early stages of STEMI with development of the adverse LV remodeling in 6 months after the event. OSM concentration in patients with STEMI at admission had also correlated with the echocardiography parameters in 6 months and widely accepted biomarkers of heart failure and myocardial necrosis at admission (Nt-porBNP, troponin I, CK-MB).

OSM is known to have multiple biological functions, including regulation of hematopoiesis, proliferation, inflammation, differentiation of mesenchyme stem cells, apoptosis and various tissues' regeneration [13] [14] [19]. The possible underlying mechanisms leading to the revealed between-groups differences in expression of OSM depending on ∆ESV values might be variability in infiltration of myocardium with macrophages, different kinetics of OSM release as well as different features of signal transduction through the specific receptors [25].

Increase of the circulating levels of OSM in subjects with coronary artery disease, heart failure, pulmonary arterial hypertension, atrial fibrillation has been demonstrated in a number of experimental and clinical studies [23] [26] [27] [28]. It became obvious that the data obtained for the role of OSM in heart function depend on the type of the pathological condition and are often quite contradictory.

Thus, OSM secreted by macrophages in atherosclerotic plaques may favor progression of atherosclerosis and dysfunction of the vascular endothelium [13] [18]. Besides influence on collagen production, proliferation and migration of smooth muscle cells, OSM may influence fibrosis, regulating balance between metalloproteinases and their inhibitors in connective tissue [14].

On the other hand a number of studies demonstrated a cardio protective role of OSM [10] [29]. This effect may be associated with employment of secondary messengers, associated with the specific intracellular enzymes and regulatory molecules.

The prior AMI is known to initiate processes of the structural reorganization in myocardium, leading to the development of post-infarct remodeling. Chronic subclinical inflammation, accompanied by the prolonged release of inflammatory cytokines, has a great impact on this process. The data on the involvement of OSM in heart remodeling in patients after AMI remain controversial and scarce.

In the recent work of Han et al. [30] the authors demonstrated that in experimental settings increased OSM favors myocardial healing via reduction of the cardiac fibrosis and preferable polarization of macrophages from M1 to M2 phenotype. However, the fact that these results have been obtained in mice with the follow-up period not exceeding 2 weeks complicates extrapolation of the results to the longterm outcome in men.

Kubin et al. [15] in their experimental studies also performed in mice were the first to underscore the dual role of OSM during heart remodeling following AMI. According to their results, OSM may initially play a protective role for the myocardium, inducing partial de-differentiation of cardiomyocytes, but its chronic influence, associated with the prolonged and extensive cardiomyocytes de-differentiation, may lead to the reduction of the contractile force, development of the ventricular dilation with elevated hemodynamic overload, heart failure and increased mortality [15] [25].

In our study we revealed that patients with acute primary STEMI are characterized by the significant elevation of OSM during the first 24 hours after the event, which is associated with unfavorable outcome in the long-term period. Identified positive associations between the level of OSM and widely studied biomarkers of heart injury and myocardial stress, which are currently used in the protocols for patients' follow-after STEMI, is a new finding which allows to regard OSM as a promising laboratory cardio-marker. Even though Bolognese et al. [31] demonstrated increased peak CK-MB in patients with LV remodeling, we did not reveal statistically significant differences in the initial levels of the heart injury biomarkers depending on the presence or absence of LV remodeling. The possible discrepancy of our results with the above mentioned study may be explained by the fact that measurement of the CK-MB activity employed by Bolognese et al. [31] may provide higher results, compared to the measurement of the CK-MB mass, which was performed in our study [32]. Hsu et al. [33] have demonstrated that peak CK-MB in combination with dynamic changes of BNP can be used to predict the development of LV remodeling in AMI patients. However, elevated peak CK-MB alone allowed to discriminate LV remodeling development only in non-STEMI patients, but not in STEMI patients, while BNP levels at admission were comparable both in groups with and without LV remodeling. In our study OSM appeared to be more advantageous biomarker for the prognosis of LV remodeling, as its dynamic evaluation was not required as well as its combination with other serum biomarkers was also not necessary.

### Limitations of the study

The comparatively small number of the recruited patients may be regarded as the major limitation of our study. However, all the patients included in our study, underwent rigorous screening to correspond to the strict inclusion and exclusion criteria, including admission time-frame, existing comorbidities, HF severity, etc. This makes us to believe that the obtained results may also be applicable to a larger cohort of STEMI patients. This does not exclude the necessity of a larger scale studies to be performed to verify the prognostic significance of the OSM early stage elevation in post-infarct heart remodeling.

## Conclusions

Thus, patients, who endured primary STEMI, demonstrate elevated concentrations of OSM in peripheral blood during the first 24 h after the event with its subsequent decrease in 6 months. Direct interconnections between OSM and troponin I, Nt-porBNP and CK-MB are detected. The level of OSM during the first 24 h correlates with left ventricular EF, as well as with the value of the LV ESV increase in 6 months after AMI. We have demonstrated that elevated levels of the biochemical marker OSM is associated with the development of the adverse left ventricular remodeling in the long-term post-infarction period and may be used to increase the efficiency of the laboratory diagnostics in patients, who experienced the primary STEMI.

## Dodatak

### Conflict of interest statement

All the authors declare that they have no conflict of interest in this work.

### List of abbreviations 

CAD, coronary artery disease;<br>CK-MB,creatine phosphokinase cardiac specific isoenzyme MB;<br>ESV,end systolic volumes;<br>LV, left ventricular;<br>Nt-proBNP, N-terminal fragment of brain natriuretic peptide;<br>OSM, oncostatin M;<br>STEMI, myocardial infarctionwith ST-elevated segment.
